# Proteome‐Wide and Immune Cell Phenotype Mendelian Randomization Highlights Immune Involvement in Genetic Generalized Epilepsy

**DOI:** 10.1002/brb3.70625

**Published:** 2025-06-10

**Authors:** Jianxiong Gui, Hongyuan Chu, Junjiao Zhang, Xiao Li, Wenwei Liu, Renqiuguo Li, Fan Zhang, Meiyu Dong, Kai Gao, Huaxia Luo, Yuwu Jiang

**Affiliations:** ^1^ Children's Medical Center Peking University First Hospital Beijing China; ^2^ Beijing Key Laboratory of Molecular Diagnosis and Study on Pediatric Genetic Diseases Beijing China; ^3^ Children Epilepsy Center Peking University First Hospital Beijing China; ^4^ Key Laboratory for Neuroscience, Ministry of Education/National Health and Family Planning Commission Peking University Beijing China; ^5^ Center of Epilepsy Beijing Institute for Brain Disorders Beijing China

**Keywords:** genetic generalized epilepsy, Mendelian randomization, plasma proteins

## Abstract

**Introduction:**

Genetic generalized epilepsy (GGE) involves polygenic inheritance, with emerging evidence implicating immune mechanisms in seizure pathogenesis. Unlike previous studies focusing on inflammation following seizures, we employed an integrative multi‐omics approach to identify precipitating immune factors in GGE development.

**Methods:**

Summary data on plasma protein levels were extracted from two large protein quantitative trait loci (pQTLs) studies, measuring 4907 and 2923 plasma proteins in 35,559 and 54,219 individuals, respectively. Immune cell trait data were derived from a genome‐wide association study (GWAS) involving 3757 individuals. GGE data, comprising 7407 cases and 52,538 controls, were sourced from a GWAS meta‐analysis by the International League Against Epilepsy (ILAE). Mendelian randomization (MR) analysis identified associations between plasma proteins, immune cell phenotypes, and GGE. Colocalization analysis assessed whether plasma proteins and GGE share a common causal variant. Transcriptome‐wide association studies (TWAS) from GTEx v8 brain tissue and whole blood were conducted for validation. Drug target prediction and molecular docking identified potential therapeutic interventions.

**Results:**

We identified 62 potential susceptibility proteins by integrating GWAS data for GGE and its subsyndromes with plasma proteomics data. Of these, eight proteins showed strong evidence of colocalization, primarily within immune‐related pathways. The absolute count of TD CD4^+^ cells was significantly associated with GGE (OR [95% CI]: 0.69 [0.59, 0.81]). Seven genes (*CD46*, *ITGAM*, *PRPSAP2*, *PYDC1*, *STX4*, *TMEM106A*, and *VAT1*) were significantly associated with GGE in at least one brain tissue in TWAS analysis. Drug target prediction and molecular docking identified several natural compounds (quercetin, cholecalciferol, resveratrol, curcumin, epigallocatechin gallate, and vitamin E) that may provide ideas for the intervention of GGE.

**Conclusion:**

These findings revealed causal associations between plasma proteins and GGE, prioritized immune‐related biological pathways, and proposed potential therapeutic hypotheses targeting immunomodulatory mechanisms.

## Graphical abstract

Genetic generalized epilepsy (GGE) accounts for >15% of all epilepsy types, however, the underlying mechanisms remain poorly understood. The current study revealed the genetic causal associations between plasma proteins and GGE. Furthermore, identified and prioritized immune‐related biological pathways. Also, proposed potential therapeutic hypotheses targeting key immunomodulatory mechanisms.

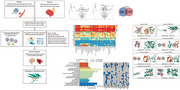

**Abbreviations**
ASMs: antiseizure medications
CAE: childhood absence epilepsy
CIs: confidence intervals
eQTLs: expression quantitative trait loci
FDR: false discovery rate
FS: febrile seizure
GGE: genetic generalized epilepsy
GTCSA: generalized tonic‐clonic seizures alone
GWAS: genome‐wide association studies
IVs: instrumental variables
IVW: inverse‐variance weighted
JAE: juvenile absence epilepsy
JME: juvenile myoclonic epilepsy
LD: linkage disequilibrium
MR: Mendelian randomization
ORs: odds ratios
PP: posterior probability
pQTLs: protein quantitative trait loci
SNPs: single nucleotide polymorphisms
TWAS: transcriptome‐wide association studies
UKB‐PPP: UK Biobank Pharma Proteomics Project

## 1 Introduction

Genetic generalized epilepsy (GGE) accounts for 15%–20% of all epilepsy types, with most cases beginning in childhood or adolescence (Jallon and Latour 2005). The classic GGE subsyndromes include childhood absence epilepsy (CAE), juvenile absence epilepsy (JAE), juvenile myoclonic epilepsy (JME), and generalized tonic‐clonic seizures alone (GTCSA) (International League Against Epilepsy Consortium on Complex Epilepsies 2023). Although these subsyndromes differ in age of onset and seizure types, their characteristics frequently overlap and may transition from one subtype to another over time. This has sparked debate about whether GGE subsyndromes should be considered distinct entities or varying manifestations within a neurobiological continuum (Vorderwülbecke et al. 2021). Family studies first provided evidence supporting the genetic etiology of GGE (Winawer et al. 2005), and it is now widely accepted that GGE follows polygenic or complex inheritance patterns rather than monogenic inheritance (International League Against Epilepsy Consortium on Complex Epilepsies 2014). Genome‐wide association studies (GWAS) have identified several genetic loci associated with GGE (International League Against Epilepsy Consortium on Complex Epilepsies 2023), but individual polymorphisms confer only modest risk, while combinations of multiple polymorphisms significantly increase susceptibility. Despite these advances, the genetic basis and underlying mechanisms of GGE remain poorly understood.

Antiseizure medications (ASMs) are the first‐line treatment for GGE; however, about 15% of patients fail to achieve seizure freedom despite treatment with two appropriate, adequately dosed ASMs (Cerulli Irelli et al. 2020). This therapeutic resistance highlights the need for alternative approaches to improve outcomes. Increasing evidence suggests that immune system dysfunction may contribute to epilepsy pathogenesis and treatment resistance. For example, levels of SEPT7, a protein associated with T‐cell migration and abnormal activation (Degroote et al. 2014), negatively correlate with drug resistance, disease duration, and seizure frequency, implicating immune mechanisms in epilepsy onset and resistance (Ayanoglu et al. 2024). Vezzani et al. showed that proinflammatory cytokines can increase neuronal excitability, exacerbating seizure susceptibility and frequency in experimental models of epilepsy (Vezzani et al. 2011). In addition, the literature suggested that genetic variations in genes associated with inflammation may contribute to predisposition to GGE (Chaves et al. 2020). Another study found that the rs16944 TT genotype, which may lead to higher IL‐1β levels, is associated with the development of epilepsy (Leal et al. 2018). However, studies on the immunogenetic factors in GGE remain limited.

Mendelian randomization (MR) has recently emerged as a valuable method for identifying potential causal relationships between exposure factors (e.g., phenotypes, plasma proteins) and disease outcomes (Skrivankova et al. 2021). MR uses genetic variants, typically single nucleotide polymorphisms (SNPs) from GWAS, as instrumental variables (IVs) to infer causality, minimizing the confounding biases of observational studies. This approach has been successfully employed to link protein quantitative trait loci (pQTLs) and identify therapeutic targets in diseases such as multiple sclerosis (Lin et al. 2023), type 2 diabetes (Yuan et al. 2023), and inflammatory bowel disease (Chen et al. 2023). However, the application of MR in GGE research, particularly in identifying pathogenic protein targets, remains limited.

In this study, we aimed to investigate the causal relationship between plasma proteins and GGE, with a focus on immune mechanisms in GGE genetic predisposition. Immunocyte phenotypes were also used in MR analysis to explore their relationship with GGE. We then conducted transcriptome‐wide association studies (TWAS) using expression quantitative trait loci (eQTLs) from GTEx v8 brain tissue and whole blood to validate our findings. In addition, potential therapeutic targets were identified through drug target prediction and molecular docking.

## 2 Methods

### 2.1 Study Design

**FIGURE 1:**
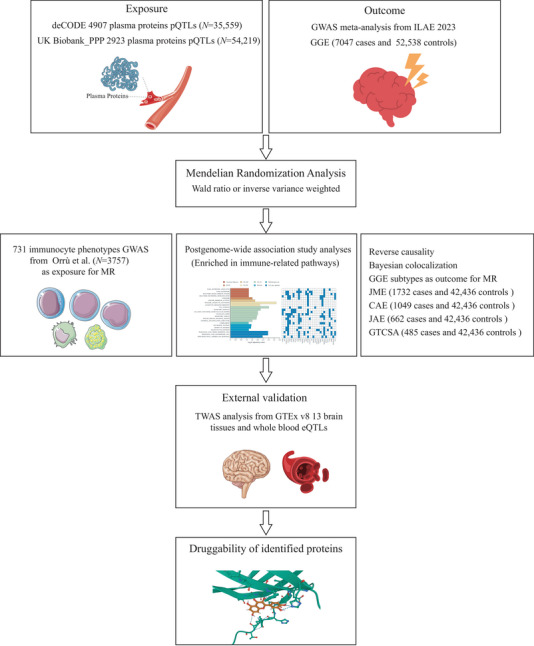
The study flowchart.

This observational study employed two‐sample MR to assess causal relationships, as depicted in Figure 1. All data were sourced from publicly available GWAS with prior ethical approval, and no experimental interventions were involved. The analyzed populations, primarily of European ancestry, followed the inclusion and exclusion criteria as specified in the original studies. The study adhered to STROBE‐MR guidelines, as outlined in Table S1.

### 2.2 Data Sources

We used data from two large‐scale GWAS on plasma protein levels: the deCODE Health Study and the UK Biobank Pharma Proteomics Project (UKB‐PPP). The deCODE study identified pQTLs for 4907 proteins in 35,559 Icelanders using the SomaScan version 4 assay (Ferkingstad et al. 2021). While the UKB‐PPP provided pQTLs for 2923 proteins from 54,219 participants, measured with the Olink Explore 3072 platform (Sun et al. 2023). pQTLs were selected according to predefined criteria (Bourgault et al. 2023; Ren et al. 2023) (Table S2). GWAS data for immunocyte phenotypes (Orrù et al. 2020) and GGE (including its subsyndromes) (International League Against Epilepsy Consortium on Complex Epilepsies 2023) were obtained from previously published studies. Additional details are provided in Table S2.

### 2.3 Mendelian Randomization Study

We conducted MR analyses via the “TwoSampleMR” package in R (version 4.3.0). The Wald ratio method was applied when only a single SNP was available as an IV, whereas the inverse‐variance weighted (IVW) method was mainly used when two or more SNPs were selected as IVs. For sensitivity analyses, we employed MR‐Egger regression, weighted median, weighted mode, simple mode, and contamination mixture methods to assess the robustness of our findings (Bourgault et al. 2023). Horizontal pleiotropy was assessed via the MR‐Egger intercept test, and heterogeneity was evaluated using Cochran's *Q* test in both IVW and MR‐Egger analyses. When Cochran's *Q* test (for heterogeneity) and MR‐Egger intercept test (for pleiotropy) yielded nonsignificant results (*p* ≥ 0.05), the IVW estimates were adopted as valid. Otherwise, sensitivity analyses employing pleiotropy‐robust methods were utilized to mitigate potential bias. Odds ratios (ORs) and 95% confidence intervals (CIs) were calculated for the associations between proteins and outcomes. To account for multiple comparisons, a 5% false discovery rate (FDR) correction was applied using the Benjamini–Hochberg (BH) procedure (Benjamini and Hochberg 1995), which offers a balanced control of sensitivity and specificity and is particularly robust for large‐scale exploratory analyses (Glickman et al. 2014) where more conservative methods, such as Bonferroni correction, may overlook biologically meaningful associations.

### 2.4 Colocalization Analysis

Colocalization analysis was performed to determine whether the genetic variants influencing plasma proteins and GGE (or its subsyndromes) share a common causal variant. The theoretical assumptions are detailed in Table S2. The analysis calculates the posterior probabilities (PPs) for each hypothesis, with the PP for H4 (PPH4) indicating the strength of evidence for colocalization (PPH4 ≥ 0.8: strong evidence for colocalization) (Yuan et al. 2023). The “coloc” package in R was used to conduct the analyses.

### 2.5 Post Proteome‐Wide Association Study Analyses

We employed the GENE2FUNC tool within the FUMA GWAS online analysis (Watanabe et al. 2017, 2019) (https://fuma.ctglab.nl/) for tissue‐specific gene expression and functional enrichment analyses of genes corresponding to proteins (FDR < 0.05) in the MR analysis. The mean expression levels of these protein‐coding genes, log2 transformed, were analyzed across 54 human tissues from GTEx v8 and visualized as a heatmap. Gene enrichment analysis identified potential pathways clustered around these genes. In addition, GeneMANIA (http://genemania.org/) was utilized to construct interaction networks centered around these genes (Franz et al. 2018).

### 2.6 Transcriptome‐Wide Association Study

We conducted TWAS using FUSION to integrate eQTLs from 13 brain tissues and whole blood in GTEx v8 with GGE GWAS data (Gui et al. 2024; Gusev et al. 2016). This approach aimed to examine associations between *cis*‐regulated genes and GGE. Following FUSION guidelines, we implemented the pipeline with default settings for TWAS. FUSION utilizes various predictive models—including top1, GBLUP, LASSO, Elastic Net, and BSLMM—to assess the combined influence of SNPs on gene expression weights. FDR correction was applied to adjust for multiple testing.

### 2.7 Potential Drug Target Prediction and Molecular Docking

We integrated MR analysis with tissue‐specific TWAS results and identified drug targets for seven proteins using the Drug Signatures Database (https://dsigdb.tanlab.org/DSigDBv1.0). We prioritized compounds with minimal side effects and potential immunomodulatory properties, including quercetin, cholecalciferol, resveratrol, curcumin, vitamin E, and epigallocatechin gallate. These compounds were further evaluated through molecular docking and binding energy analysis.

First, we retrieved the molecular structures of candidate drugs from the PubChem compound database (https://pubchem.ncbi.nlm.nih.gov/) (Wang et al. 2017). The structures of the proteins were downloaded from the Protein Data Bank (PDB) (http://www.rcsb.org/) and predicted using AlphaFold (https://alphafold.ebi.ac.uk/) (Varadi et al. 2023) (Table S2). Protein and ligand files were prepared by converting them into PDBQT format, removing water molecules, and adding polar hydrogens. The grid box was centered to cover the protein domains and allow for free molecular movement, with the binding pocket set to a cubic space of 30 Å × 30 Å × 30 Å and a grid spacing of 0.05 nm. Binding energy, interaction modes, and visualizations of the candidate drugs with their target molecules were analyzed using AutoDock Vina v1.2.2 (http://autodock.scripps.edu/) (Morris et al. 2008).

## 3 Results

### 3.1 Proteome‐Wide Mendelian Randomization Studies of GGE

**FIGURE 2:**
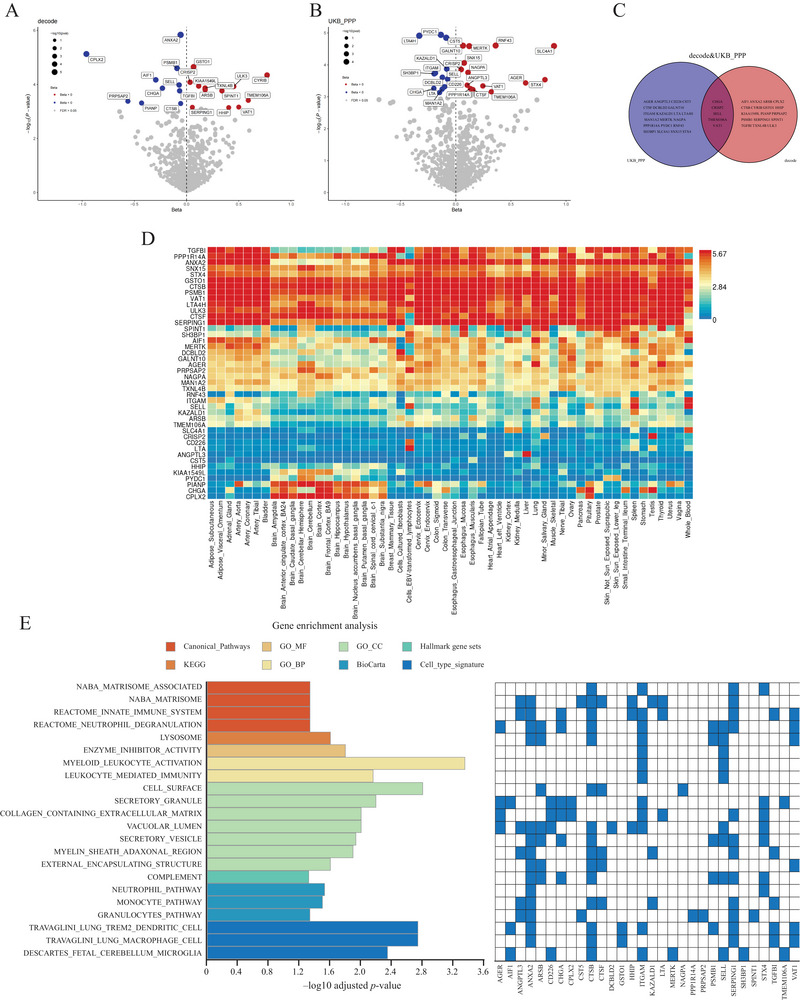
Proteome‐wide MR studies of GGE. (A) Volcano plot from the proteome‐wide MR study of GGE using deCODE data. (B) Volcano plot from the proteome‐wide MR study of GGE using UKB‐PPP data. Annotated proteins represent significant associations with GGE after FDR correction (FDR < 0.05). Blue and red colors indicate negative and positive effects, respectively. (C) Venn diagram showing proteins associated with GGE in deCODE only, UKB‐PPP only, or both. (D) Heatmap of gene expression for 43 unique significant proteins from proteome‐wide MR in deCODE and UKB‐PPP. Log2 transformed average expression across 54 GTEx v8 tissues. Red indicates higher expression levels, while blue indicates lower expression levels. (E) Pathway analysis of the 43 unique proteins identified in deCODE and UKB‐PPP. Pathways with significant enrichment (FDR < 0.05) and overlap of at least two genes are presented.

To identify plasma proteins associated with GGE, we performed proteome‐wide MR analyses using two large datasets. A total of 1510 proteins from the deCODE study and 1707 proteins from the UKB‐PPP study passed the selection criteria. After applying a 5% FDR correction for multiple tests, MR analysis identified 22 proteins in deCODE (Figure 2A) and 26 proteins in UKB‐PPP (Figure 2B), with five proteins overlapping between both datasets (CHGA, CRISP2, SELL, TMEM106A, and VAT1) (Figure 2C). Egger intercept and Cochran's *Q* test indicated minimal pleiotropy and heterogeneity, supporting the reliability of the IVW results (Table S3a,b). Colocalization analysis revealed strong evidence of shared causal variant between five proteins and GGE: ANXA2 (PPH4_decode_ = 0.97), CPLX2 (PPH4 _decode_ = 0.92), SLC4A1 (PPH4_UKB‐PPP_ = 0.94), STX4 (PPH4_UKB‐PPP_ = 0.92), and LTA (PPH4_UKB‐PPP_ = 0.82) (Figure S1). Moderate evidence was provided for eight proteins (Table S3a,b). The F‐statistics indicated robust instrument strength.

In the reverse MR analysis, where GGE was used as the exposure and plasma proteins as the outcome, GGE was found to be significantly associated with 19 proteins after FDR correction (Table S3c). Of the 43 previously identified proteins, only KAZALD1 may have a bidirectional regulatory relationship with GGE, while the others are more likely to act as protective or risk factors.

### 3.2 Post Proteome‐Wide Association Study Analyses

To further characterize the identified proteins, we explored their tissue‐specific expression patterns and functional pathways. As shown in Figure 2D, tissue‐specific gene expression patterns were assessed for 43 proteins significantly associated with GGE. Overall, a subset of genes, including *PPP1R14A*, *ANXA2*, *SNX15*, *STX4*, *GSTO1*, *GTSB*, *PSMB1*, *VAT1*, *LTA4H*, *ULK3*, *CTSF*, and *SERPING1*, displayed consistently high expression levels across multiple tissues, suggesting their ubiquitous biological roles. In contrast, genes such as *PIANP*, *CHGA*, and *CPLX2* exhibited predominantly high expression restricted to brain tissues, highlighting their potential tissue‐specific relevance to neurological processes. We then conducted gene enrichment analysis to identify potential functions, with the highest‐ranking pathways after *p*‐value correction presented in Figure 2E. Many enriched pathways were immune‐related, including “Reactome innate immune system,” “Reactome neutrophil degranulation,” “Myeloid leukocyte activation,” “Activation leukocyte‐mediated immunity,” “Complement,” “Neutrophil pathway,” “Monocyte pathway,” “Granulocytes pathway,” and “Descartes fetal cerebellum microglia.” Given that TREM2 expression in microglia is essential for maintaining normal neuronal bioenergetics during development (Tagliatti et al. 2024), the pathways “Travaglini lung TREM2 dendritic cell” and “Travaglini lung macrophage cell” also drew our attention.

### 3.3 Immunocyte Phenotypes Mendelian Randomization Studies of GGE

**FIGURE 3:**
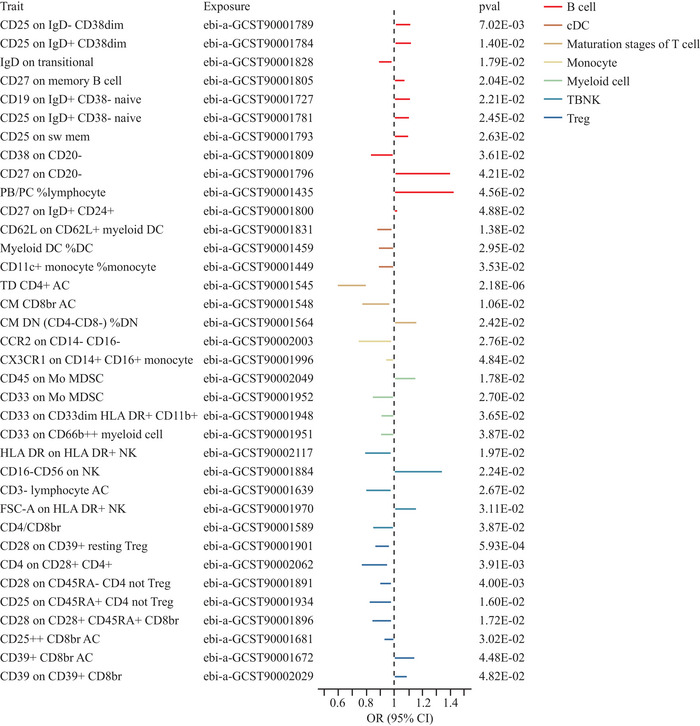
Results of MR analysis of immunocyte phenotypes and GGE (*p* < 0.05), with different panels represented by colored lines.

Given the enrichment of immune pathways, we conducted MR analyses to assess the relationship between immune cell traits and GGE. A total of 720 immunocyte phenotypes met the selection criteria for further analysis. Ultimately, 36 immune cell phenotypes were significantly associated with GGE (*p* < 0.05) (Table S4). Most B cell subsets exhibited a positive correlation with GGE, while conventional dendritic cells (cDC), T‐cell maturation stages, monocytes, myeloid cells, and regulatory T cells (Treg) displayed a negative correlation (Figure 3). After FDR correction, the absolute count of TD CD4^+^ cells was significantly associated with GGE (OR [95% CI]: 0.69 [0.59, 0.81]).

### 3.4 Mendelian Randomization and Gene Function Analysis of GGE Subsyndromes

**FIGURE 4:**
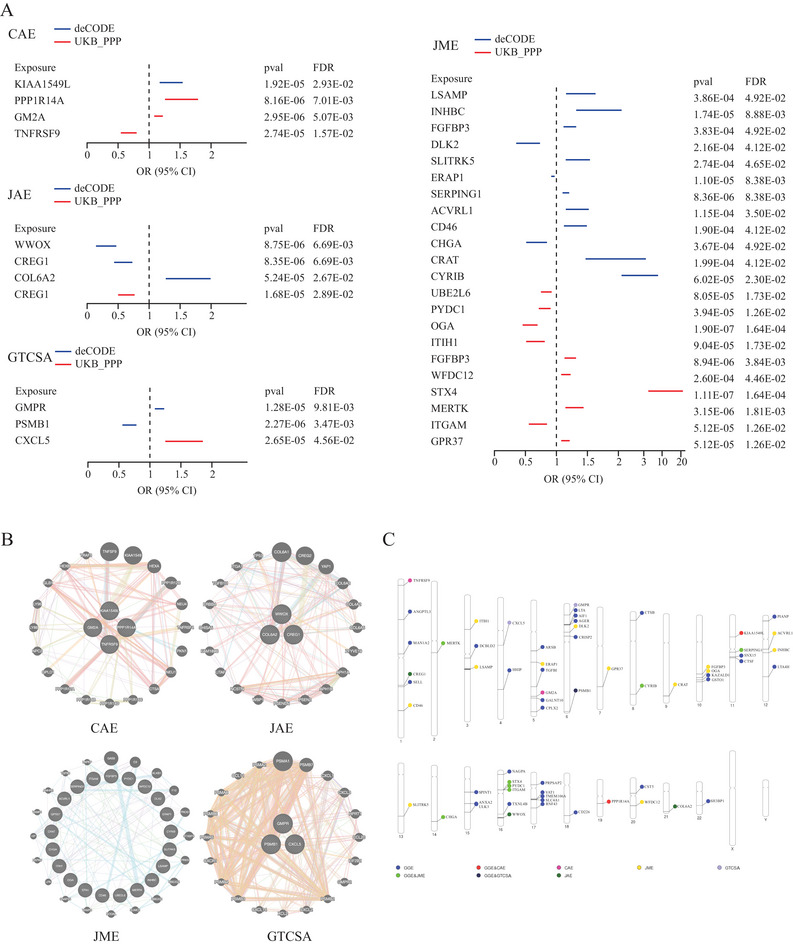
Proteome‐wide MR studies of GGE subsyndromes. (A) Causal effects of MR analysis between plasma proteins and four subsyndromes (FDR < 0.05). Blue lines represent deCODE data; red lines represent UKB‐PPP data. (B) Protein–protein interaction networks for each subsyndrome established using GeneMANIA. (C) PhenoGram showing significant associations identified in proteome‐wide MR studies of GGE and its subsyndromes. The genes encoding these proteins are located on chromosomes 1–22. Different colors represent associations with GGE, JME, CAE, GTCSA, or combinations thereof.

Recognizing the heterogeneity within GGE, we extended MR analyses to its subsyndromes, including CAE, JAE, JME, and GTCSA, to identify subtype‐specific plasma protein associations. By integrating findings from the deCODE and UKB‐PPP datasets, we identified significant associations between plasma proteins and various GGE subsyndromes. Specifically, four plasma proteins were significantly linked to CAE, four to JAE, 21 to JME, and three to GTCSA (Table S5a–h and Figure 4A). Colocalization analysis provided strong evidence for the colocalization of three proteins (SERPING1 PPH4_decode_ = 0.88, OGA PPH4_UKB‐PPP_ = 0.96, and STX4_UKB‐PPP_ = 0.94) with JME (Table S5e,f and Figure S2).

GeneMANIA was used to establish protein‐protein interaction networks for each subtype (Figure 4B). The expression level of genes corresponding to key proteins identified for each subtype was visualized across different tissues using heatmaps (Figure S3). In JME, these genes were primarily enriched in pathways related to the “complement system in neuronal development and plasticity” and the “innate immune response” (Figure S4). No significant pathways were identified in CAE, JAE, or GTCSA.

We further integrated the MR results of plasma proteins with GGE and its subsyndromes, identifying several plasma proteins that were significantly associated with both GGE and its subsyndromes, supported by colocalization evidence (Table S6). Plasma levels of STX4 and CYRIB proteins were positively associated with both GGE and JME, while ITGAM and PYDC1 showed a negative association with GGE and JME. As shown in Figure 4C, the genes encoding the 62 proteins identified in the proteome‐wide MR analysis are mapped across autosomes 1–22. These genes are widely distributed without obvious clustering on specific chromosomes, suggesting that the identified associations are not driven by localized chromosomal effects. Notably, genes associated with subsyndromes such as JME, CAE, and GTCSA are interspersed among those linked to GGE overall, supporting shared and distinct genetic underpinnings across subtypes.

### 3.5 Transcriptome‐Wide Association Study

To complement the proteome‐wide findings, we performed TWAS to uncover gene expression profiles linked to GGE. In TWAS analysis, 274 genes were significantly associated with GGE across various tissues after FDR correction (Table S7). We identified nine genes from the proteome‐wide MR analysis and selected seven of them (*CD46*, *ITGAM*, *PRPSAP2*, *PYDC1*, *STX4*, *TMEM106A*, and *VAT1*), which were significantly associated with GGE in at least one brain tissue, for further analysis.

### 3.6 Potential Drug Target Prediction and Molecular Docking

Finally, to explore therapeutic implications, we predicted potential drug targets among key proteins and conducted molecular docking analyses. Drug compounds with potential interaction of seven target genes were identified (Table S8a). Pharmacological agents with unclear mechanisms of action or those known to have significant adverse effects, such as antineoplastic drugs (e.g., paclitaxel, vincristine) were excluded. Given preliminary evidence suggesting immune‐related mechanisms in GGE development, we selected drugs with potential immunoregulatory properties—quercetin, cholecalciferol, resveratrol, curcumin, epigallocatechin gallate, and vitamin E—for further analysis. Well‐established ASMs (e.g., valproic acid, phenobarbital) were used as reference.

**FIGURE 5:**
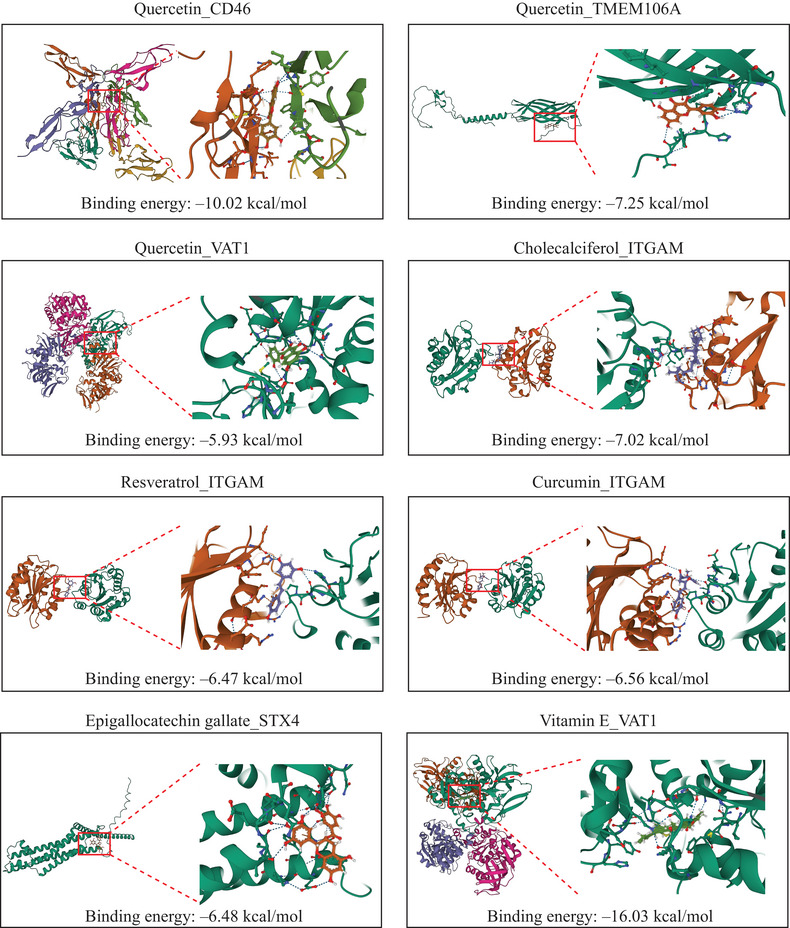
Molecular docking of screened drugs with their targets.

Using AutoDock Vina, we obtained the binding poses and interactions for six candidate drugs with five proteins, generating the binding energies for each interaction (Figure 5 and Table S8b). The results demonstrated that all candidate drugs formed observable hydrogen bonds and electrostatic interactions with their respective protein targets. Notably, vitamin E exhibited the strongest binding affinity with VAT1, showing a binding energy of −16.03 kcal/mol. In the optimal docking models, quercetin showed binding energies of −10.02, −7.25, and −5.93 kcal/mol with CD46, TMEM106A, and VAT1, respectively. Cholecalciferol, resveratrol, and curcumin demonstrated binding energies of −7.02, −6.47, and −6.56 kcal/mol with ITGAM, respectively, while epigallocatechin gallate exhibited a binding energy of −6.48 kcal/mol with STX4. Natural compounds exhibit lower binding affinities than valproic acid or phenobarbital and their respective targets (Figure S5).

## 4 Discussion

GGE is a common complex disorder in which no potential causes beyond genetic factors have been identified. Therefore, it is crucial to identify the susceptibility genes associated with GGE. The combination of plasma proteomics and MR has emerged as a powerful approach to elucidate disease mechanisms and identify potential drug targets (Chen et al. 2023; Lin et al. 2023; Yuan et al. 2023). In our study, we identified 62 potential susceptibility proteins by combining GWAS data on GGE and its subsyndromes with plasma proteomics data from deCODE and UKB‐PPP. Among these, eight proteins were supported by strong evidence of colocalization.

While the enrichment of immune‐related pathways in GGE is clear, our study does not fully address whether immune dysregulation is a cause or consequence of GGE. Several studies have implicated immune system dysfunction in epilepsy pathogenesis (Leal et al. 2018; Varadkar et al. 2014; Vezzani et al. 2011), and our MR analysis suggests that immune proteins such as ITGAM, SERPING, and PYDC1 may contribute to susceptibility. ITGAM is integral to adhesion processes in monocytes, macrophages, and granulocytes, and plays a key role in DC‐mediated T‐cell activation (Losse et al. 2010; Sim et al. 2021). SERPING1 (C1 esterase inhibitor, C1‐INH) regulates the complement system, preventing excessive complement activation, modulating inflammation, and protecting against autoimmunity (Ryø et al. 2023). PYDC1 (Pyrin domain‐containing protein 1) is involved in the activation of NF‐κB and procaspase‐1, playing a critical role in innate immunity (Stehlik et al. 2003). However, immune activation could also result from ongoing neuronal activity and seizures, raising the question of whether immune dysregulation is a consequence of GGE rather than its origin. In the case of KAZALD1, our reverse MR analysis suggested a bidirectional relationship with GGE, indicating that it could both affect and be affected by GGE. This dual role suggests that KAZALD1 may participate in a feedback loop where immune activation in response to GGE further exacerbates the disorder, while its expression could contribute to disease progression. Further studies examining the temporal relationship between immune responses and epileptic events are needed to clarify this issue.

To further explore the immunological basis of GGE susceptibility, we extended our investigation to immune cell phenotypes. Notably, our analyses revealed that the absolute count of thymus‐dependent CD4^+^ helper T cells (TD CD4^+^ AC) and Treg, a subtype of TD CD4^+^, were significantly negatively correlated with GGE. The study by Liu et al. showed that decreased T cells can correlate with increased seizures, thus strengthening the argument that the immune system, and specifically T cells, play an important role in the pathogenesis of GGE (Liu et al. 2022). In addition, B cells, dendritic cells, monocytes, and myeloid cells were closely associated with GGE, warranting further attention in future studies.

MR analysis of GGE subsyndromes revealed that CAE, JAE, JME, and GTCSA might have distinct susceptibility genes, though the possibility of inconsistent statistical power due to small sample sizes cannot be excluded. Notably, the potential susceptibility genes for JME were significantly enriched in immune response pathways, further supporting the role of immune involvement in GGE susceptibility.

Moving forward, our study aims to identify drugs targeting susceptibility genes to reduce GGE risk or aid its treatment. Through TWAS, we identified genes that have been validated as closely associated with GGE in at least one brain tissue, designating them as potential therapeutic targets. While discrepancies between plasma and brain tissue correlations with GGE for some genes—potentially due to tissue‐specific functions or compensatory mechanisms (Nonoguchi et al. 2022)—exist, they do not undermine our focus. Drug target predictions for seven genes were followed by molecular docking, selecting compounds based on proven safety and preference for naturally derived substances with potential immunoregulatory properties.

While our molecular docking analyses revealed plausible interactions between candidate compounds and target proteins, it is important to acknowledge the preliminary nature of these computational predictions. The observed binding energies (−5.93 to −16.03 kcal/mol) for natural compounds were generally weaker than typical drug‐target interactions (commonly < −8 kcal/mol), suggesting these may represent partial modulatory effects rather than high‐affinity binding. This aligns with the known polypharmacology of natural compounds, where therapeutic effects often arise through multitarget synergies rather than single high‐affinity interactions (Daina et al. 2019).

These findings gain biological plausibility from prior evidence of antiepileptic effects in natural compounds. Quercetin exhibits anti‐inflammatory and neuroprotective properties through modulation of the activation of microglia (Han et al. 2021; Li et al. 2016), while resveratrol reduces neuroinflammation via PGC‐1α pathway activation (Yang et al. 2017). Clinical observations suggest cholecalciferol (vitamin D_3_) reduces seizure frequency in drug‐resistant epilepsy (Holló et al. 2012), and animal studies support the antiepileptic effects of epigallocatechin gallate, vitamin E, curcumin, quercetin, and resveratrol, including prolonged seizure latency, reduced seizure frequency, and mitigation of seizure‐induced pathological changes (Akyuz et al. 2021; Alrashdi et al. 2023; Dhir 2018; Lu and Wang 2015; Upaganlawar et al. 2021). Although low binding energies may indicate partial target engagement, the therapeutic efficacy of these compounds likely emerges from synergistic modulation of multiple pathways. Future studies should incorporate negative control ligands and experimental validation to confirm binding specificity.

Our analysis of plasma protein‐GGE correlations provides hypothetical insights into pathophysiological pathways, suggesting that certain compounds might modulate biological processes relevant to seizure susceptibility. Previous work has demonstrated that genetically‐informed target selection could theoretically improve therapeutic development efficiency (Nelson et al. 2015). The observed association between febrile seizure (FS) history and GGE susceptibility (Mohanraj and Brodie 2007) raises questions about whether early immune modulation after FS might influence epileptogenesis, though causal relationships remain unproven. The extensive involvement of immune pathways suggests that multitarget interventions could theoretically complement existing ASMs, particularly in drug‐resistant cases. However, translational applications would require rigorous evaluation of target specificity and dose‐response relationships, given the pleiotropic nature of phytochemicals.

While prior work has characterized the immune status changes following epileptic seizures (Patel et al. 2019), our study advances the field by mapping precipitating immune mechanisms in GGE pathogenesis. Through multi‐omics integration of plasma proteomics and brain transcriptomics, we identified potential susceptibility loci (e.g., ITGAM, SERPING1) bridging innate immunity with neuronal hyperexcitability. The cross‐subsyndrome analysis further revealed divergent immune signatures between JME and other subtypes, suggesting previously unrecognized etiological heterogeneity. Our methodological rigor—combining MR causality testing, TWAS validation, and drug target prioritization—provides a framework for transforming observational associations into therapeutic hypotheses with biological plausibility. To advance these findings, three key directions emerge: (1) functional validation of susceptibility genes, (2) clinical evaluation of prioritized compounds through adaptive trial designs, and crucially, (3) longitudinal profiling of plasma proteome and immunology over time in individuals with GGE.

However, several limitations must be acknowledged. Due to the selection criteria for IVs, some pQTLs corresponding to proteins were excluded from the analyses. Consequently, only 1510 proteins (deCODE) and 1707 proteins (UKB‐PPP) were analyzed, which is relatively small compared to the full spectrum of detectable human proteins, potentially omitting many susceptibility genes. The limited overlap of only five proteins identified across both cohorts, likely attributed to platform heterogeneity or population stratification, may compromise the reproducibility of the findings. As larger‐scale proteomics studies emerge, this limitation will likely be addressed. In addition, the limited number of GGE subsyndrome cases hindered significant pathway enrichment for the susceptibility genes identified in CAE, JAE, and GTCSA, complicating the exploration of differences between subsyndromes. However, the generalizability of our findings may be constrained by their derivation from European population data, thus requiring validation in ethnically diverse cohorts to ensure broader applicability. Furthermore, although we predicted potential drug interactions for some targets, we only validated molecular docking for a few natural compounds. While these compounds have shown efficacy in animal models of epilepsy, the models used involve specific seizure types that differ from the pathogenic process of GGE. Lastly, these findings require validation through basic experiments or clinical cohorts and should be interpreted with caution.

## 5 Conclusion

In conclusion, this study employed integrated genetic approaches to identify multiple associations between plasma proteins and the risk of GGE and its subsyndromes. Enrichment analysis and MR analysis of immune cell phenotypes suggest that genetic factors related to immunity may contribute to the pathogenesis of GGE. Drug target prediction suggests that certain natural compounds with immunomodulatory properties may provide ideas for exploring the treatment of GGE. These findings provide new insights into the pathogenic mechanisms and possible therapeutic targets for GGE, though further validation is required.

## Author Contributions

**Jianxiong Gui**: conceptualization, methodology, software, data curation, writing – original draft, writing – review and editing, visualization, validation, investigation. **Hongyuan Chu**: conceptualization, methodology, writing – review and editing, writing – original draft. **Junjiao Zhang**: writing – review and editing. **Xiao Li**: visualization. **Wenwei Liu**: data curation. **Renqiuguo Li**: methodology. **Fan Zhang**: validation. **Meiyu Dong**: validation. **Kai Gao**: writing – review and editing. **Huaxia Luo**: methodology. **Yuwu Jiang**: conceptualization, methodology, supervision, writing – review and editing.

## Ethics Statement

The authors have nothing to report.

## Consent

The authors have nothing to report.

## Conflicts of Interest

The authors declare no conflicts of interest.

## Peer Review

The peer review history for this article is available at https://publons.com/publon/10.1002/brb3.70625

## Supporting information




**Supporting Figures**: brb370625‐sup‐0001‐FigureS1‐S5.docx

**Supporting Tables**: brb370625‐sup‐0002‐Tables.xlsx

## Data Availability

Publicly available datasets were analyzed in this study. These datasets can be found at the following URLs: deCODE (https://www.decode.com/summarydata/); UKB‐PPP (https://www.synapse.org/Synapse:syn51365301); GWAS Catalog (https://www.ebi.ac.uk/gwas/downloads/summary‐statistics); GWAS summary data of GGE and its subsyndromes (https://www.epigad.org). Any data generated during the study process are available for reasonable requests by emailing Jianxiong Gui (gui199508046@163.com).
