# Supplementary material for: Proteome‐Wide and Immune Cell Phenotype Mendelian Randomization Highlights Immune Involvement in Genetic Generalized Epilepsy
Source: Brain Behav. 2025 Jun 10;15(6):e70625. doi: 10.1002/brb3.70625 (PMC12152256; doi:10.1002/brb3.70625)
Supplement: Supplementary file 1 — Supporting Figures: brb370625‐sup‐0001‐FigureS1‐S5.docx [file BRB3-15-e70625-s002.docx]

**Supplementary** **Figures**

**Figure S1.** The results of colocalization analysis between plasma proteins and GGE (PPH4≥0.8).


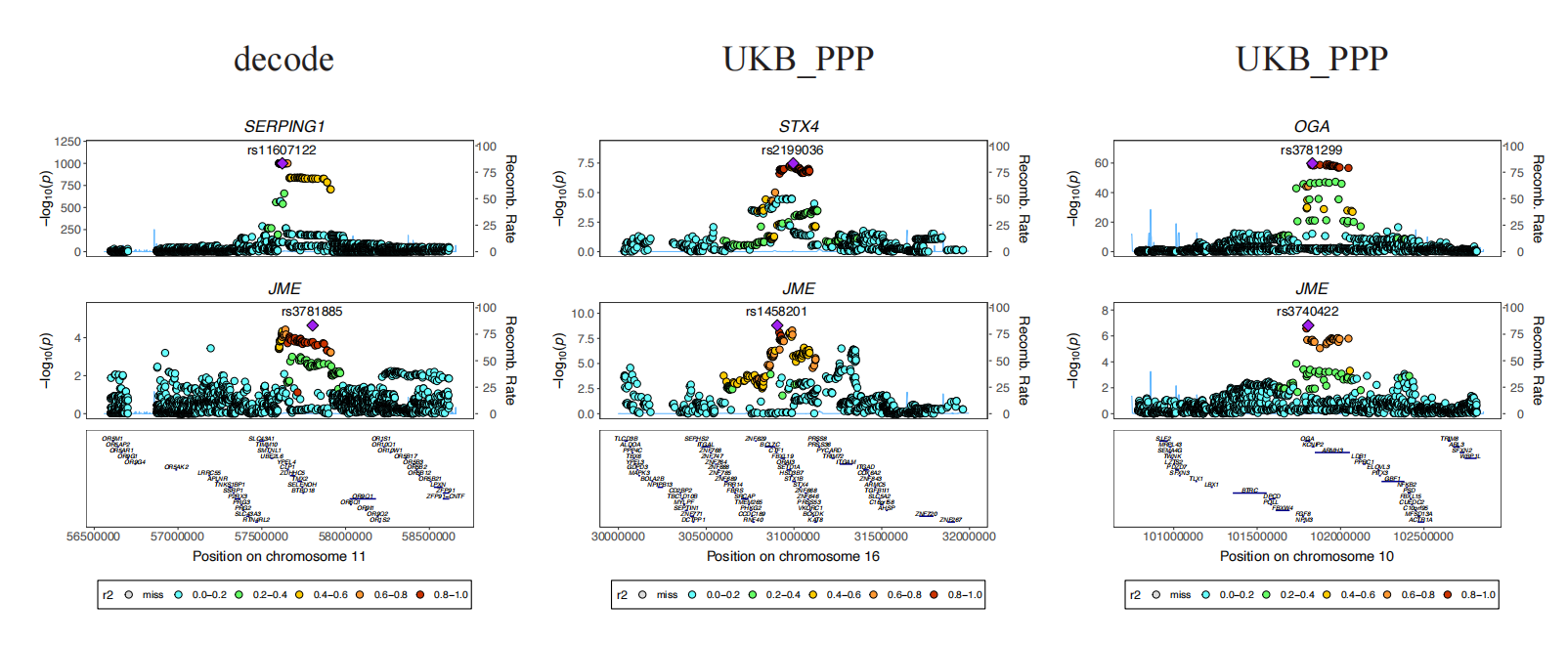


**Figure S2.** The results of colocalization analysis between plasma proteins and JME (PPH4≥0.8).

**Figure S3.** Heat map of gene expression for significant proteins from Proteome-Wide MR in CAE, JAE, GTCSA and, JME. Log_2_-transformed average expression across 54 GTEx V8 tissues.

**Figure S4.** Pathway analysis of the significant proteins with JME. Pathways with significant enrichment (FDR < 0.05) and overlap of at least two genes are presented.
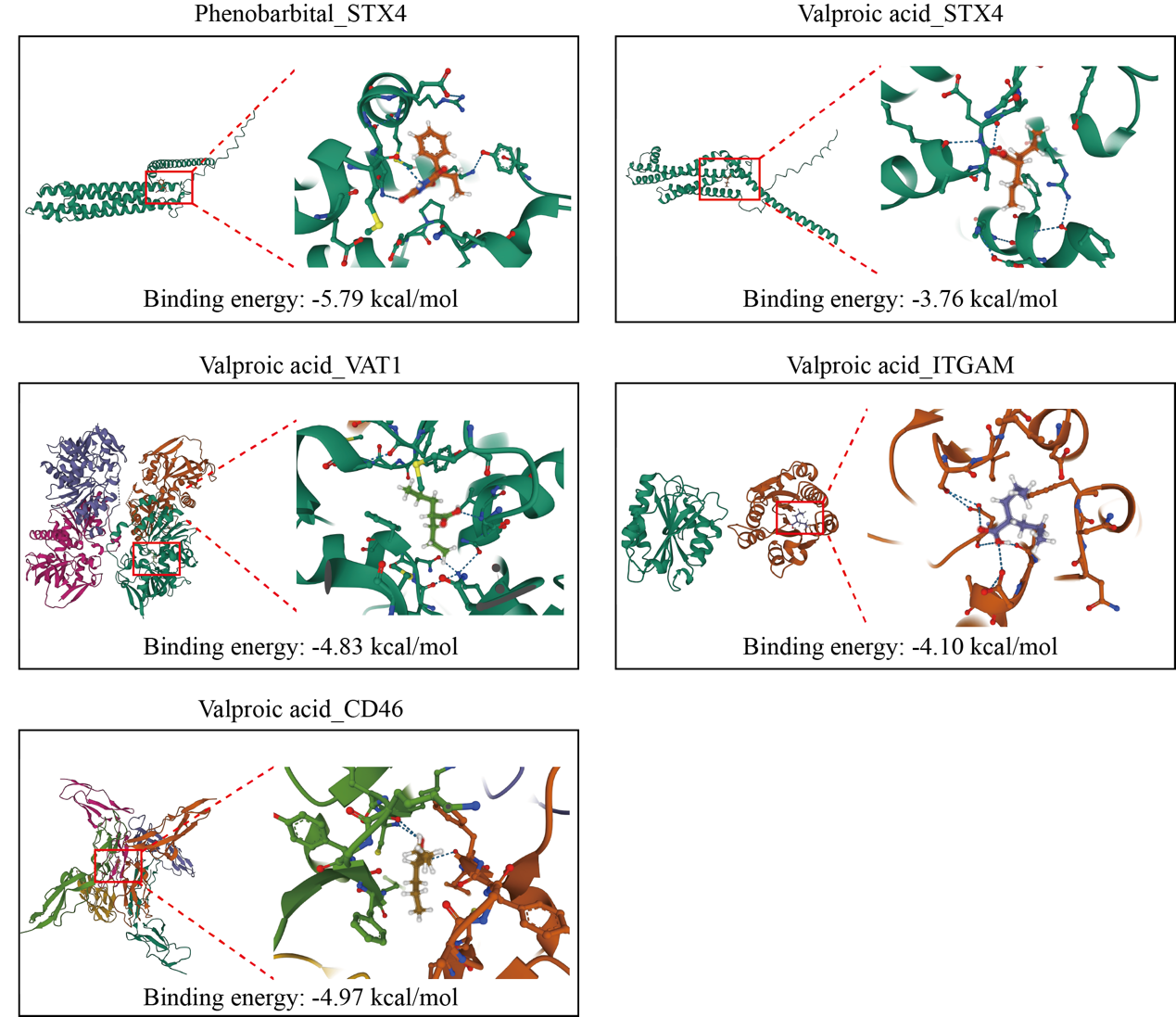


**Figure S5.** Molecular docking of ASMs with their targets.
